# Supplementary material for: Are carbon emissions trading and green financial instruments synergistic? -Comprehensive quantitative research based on content analysis
Source: PLoS One. 2024 Mar 7;19(3):e0298601. doi: 10.1371/journal.pone.0298601 (PMC10919601; doi:10.1371/journal.pone.0298601)
Supplement: S1 Data — (ZIP) [file pone.0298601.s001.zip › Data/地级市Z_F工作报告.zip!/║■─╧/╥μ╤⌠╩╨/└┤╫╘╣1⁄2╓┌║┼í╛┬φ┐╦╩2╛▌═°í┐_2018.docx]

各位代表：

现在，我代表市人民政府向大会作工作报告，请予审议，并请各位政协委员和其他列席人员提出意见。

2017年工作回顾

2017年是新一届政府的开局之年，也是极不平凡的一年。一年来，在省委、省政府和市委的正确领导下，我们主动适应经济发展新常态，坚持稳中求进工作总基调，大力实施“创新引领、开放崛起”战略，有力应对严重洪涝灾害等重大挑战，着力打好脱贫攻坚、转型升级和环境治理“三大战役”，经济发展保持稳中有进、稳中向好、稳中趋优的良好态势，主要经济指标增速高于全省平均水平，排位整体前移。全市实现生产总值1666亿元（预计数，下同），增长8.2%；完成固定资产投资1501亿元，增长14.1%；规模工业增加值增长7.5%；实现社会消费品零售总额711亿元，增长10.8%；完成一般公共预算收入116.8亿元，增长9%；实现全体居民人均可支配收入20077元，增长9.5%。全面小康总体实现程度达到90%。

一、着力推进产业建设，转型升级步伐加快

工业经济企稳向好。企业帮扶“六大专项行动”取得成效，新增规模企业120家，新增湖南名牌产品31个。完成工业技改投入531.7亿元，增长8%；工业税收和工业用电量分别增长25%、10.5%。园区建设大会战扎实推进，完成基础设施投资122.8亿元，新建标准化厂房106.7万平方米，利用率达84.4%；新开工、新投产项目分别达137个、124个，园区规模工业增加值占比达71.3%，提高2.1个百分点。农业生产质效提升。实现农林牧渔业总产值485.7亿元，增长3.6%；稻虾、稻鱼、稻蟹等高效生态种养面积达45万亩，平均每亩增收2000元以上。举办首届生态农业智慧乡村互联网大会，推动互联网与“三农”融合发展。创建现代农业产业园、科技园、创业园22个，新增“三品”认证138个。土地适度规模经营比率达52%。安化黑茶成为中欧国际互认地标产品，南县小龙虾、桃江竹笋、沅江芦笋和四季红镇腐乳获得国家级登记认证。现代服务业加快发展。开展国家旅游休闲示范城市创建，大力发展全域旅游，举办首届洞庭湖生态文化旅游暨水上运动节、中国·湖南（南县）首届国际涂鸦艺术节等活动，云梦方舟、万达广场、海洋城等建成运营。实现旅游综合收入235.8亿元，增长60.3%。现代金融体系不断完善，金融机构各项存贷款余额比年初分别增长10.1%和24.5%，新增贷款190亿元。政策性农保覆盖面进一步扩大。奥士康成功上市，创今年全省单个IPO项目融资最高纪录。电子商务快速发展，全市电商达1.8万余家（户），线上交易额突破300亿元，快递出港量增长15%以上。安化获批全国电子商务进农村综合示范县。兰溪粮食物流园、安化黑茶物流中心等项目加速推进。房地产投资和商品房销售面积分别增长10%和15%。开放创新亮点纷呈。积极开展“五大开放行动”，举办首届“汇智聚力·建设益阳”恳谈会等系列活动，签约重点项目79个。与中车、华为、中机、航天科工等央企和行业龙头开展对接合作。中以合作示范城市创建纳入国家层面。引进市域外资金形成固定资产投资453亿元，增长15%。完成进出口额49亿元，增长16%，新增外贸破零企业30家。长沙海关驻益阳办事处和益阳出入境检验检疫机构正式运行。加大创新创业政策支持力度，评选表彰科技、产品、文化、管理四大创新奖29项。高新技术产业实现增加值315亿元，增长16%。申请发明专利824件，新增省级科技创新平台16家。“互联网＋”行动深入推进，互联网与经济社会各领域融合发展进一步深化。

二、着力推进基础设施建设，支撑能力逐步增强

深入推进项目建设年活动，开展项目建设大开工、大推进、大攻坚、大会战和重点项目巡回观摩点评，完善重点项目调度机制。全市施工项目和亿元以上项目比上年分别增加206个、72个。交通建设大会战强力推进。完成交通建设投资155.5亿元，增长16.4%。益娄高速提前一年通车，益马高速基本建成，马安、南益、长益复线高速加快建设，官新、伍益、宁韶高速顺利开工。干线公路建设“519”行动完成16个项目235公里，新建农村公路1432公里，打通了一批边界路、瓶颈路。石长铁路动车正式开通，常益长高铁已开工建设，现代综合交通体系正在逐步形成。水利基础设施不断改善。水利秋冬修和水毁工程修复扎实开展，完成投资24.5亿元，修建各类水利工程5.5万处。农饮巩固提升工程稳步推进，解决了5.9万建档立卡贫困人口饮水安全问题，农村自来水普及率达83.5%。能源和信息基础设施建设全面加强。大通湖渔光互补光伏发电、沅江华顺漉湖风电场等新能源项目建成投产。464个自然村完成农网改造。气化工程加速推进，中心城区天然气普及率达60%以上。国家电信普遍服务试点市项目顺利完成，“宽带中国”示范城市建设扎实推进。

三、着力推进城乡建设，区域发展更加协调

益沅桃城镇群建设大力推进。益沅桃城镇群被确定为全省第一批产城融合综合试点。完成城镇基础设施建设投资282.1亿元，资江风貌带、一园两中心、西流湾大桥、青龙洲大桥、益阳大道西延线等重点项目进展顺利。全国文明城市创建工作扎实开展，国家卫生城市复审顺利通过。城市规划区山体水体保护工作全面启动。垃圾减量化、无害化、资源化处理“益阳模式”在全省推广。数字城管平台投入运行。城市“双修”扎实推进，完成7处易涝点治理、9处黑臭水体整治和894个“三微”项目建设，解决了一批人民群众关切的热点难点痛点问题。东接东进稳步推进。重点在产业、交通等方面加强与长沙对接。东部片区基础设施不断完善，长益高速湘江西互通建成通车，东接东进门户大步前移。完成高新大道、智慧谷路、鱼形山路提质改造。鑫兴嘉德、愿景住工等产业项目竣工投产，量子动力、瑞思光学等一批项目落户东部片区。县城和重点镇建设扎实推进。加强县城和国家重点镇、省级新型城镇化试点镇及市级示范镇建设，兰溪镇、长春镇、小淹镇、厂窖镇获评省级美丽乡镇。美丽乡村建设有序推进。持续实施农村清洁工程，深入开展美丽乡村示范村创建，着力打造资阳紫薇村、南县罗文村、赫山菱角岔村等美丽乡村示范村（片）。桃江朱家村获评中国美丽休闲乡村，安化九龙池村、桃江花园洞村入选第四批中国传统村落。

四、着力推进生态建设，人居环境持续优化

狠抓环保督察问题整改。加大环保执法和问责力度，中央环保督察交办信访件156件全部办结，省环保督察反馈问题整改同步推进。立案查处违法违规企业190家，行政拘留28人、刑事拘留5人，172名党员干部被问责。全面推行河长制。完成全市河湖名录摸底造册，明确市县乡村四级河长，实现所有河流湖泊工作责任全覆盖，“三长一站”模式在全省推广。强力推进环境综合治理。扎实开展洞庭湖水环境综合治理五大专项行动。建成7个集镇和8个工业园区污水处理厂。完成河道清理、沟渠疏浚、塘坝清淤年度任务。关停、搬迁规模养殖户3448户。强力推进“两围两网”整治，彻底拆除沅江下塞湖节制闸，全面完成河湖围网清理任务。南洞庭湖湿地核心区黑杨全部清除。中心城区饮用水源问题整治到位，资水益阳段总体水质为优，柘溪水库水质由Ⅲ类改善为Ⅱ类，大通湖总磷指标持续下降。重金属污染治理、矿山复绿和生态修复取得进展。新增新能源公交车400台，淘汰黄标车6947台，关闭粘土砖厂145家，大气二氧化硫含量比上年下降50%以上，空气质量明显改善。

五、着力推进民生建设，民生福祉不断增进

脱贫攻坚扎实有效。完成1.47万人易地扶贫搬迁，发放扶贫小额贷款6.6亿元，退出贫困村141个，8.5万人口脱贫。就业创业成效明显。新增市场主体4.3万户。新增城镇就业3.4万人、农村劳动力转移就业1.8万人。社保体系不断完善。完成全民参保登记计划，实现基本养老、医疗保险全覆盖。农村低保和扶贫标准实现“两线合一”，1.7万名农村建档立卡贫困对象提前实现兜底保障。改扩建乡镇敬老院26所。大力推进保障性住房建设和农村危房改造，解决16.1万住房困难群众安居问题。城乡特困救助、移民避险解困、留守儿童保护、残疾人关爱、希望工程等工作统筹推进。社会事业协调发展。推进教育强市建设，新建学校10所，改扩建学校71所，建设合格学校59所、农村公办幼儿园21所。启动中心城区“四建五促十二扩”工程，大班额化解工作取得实效。启动市级公费师范生培养计划，定向培养乡村教师319名。倡导尊师重教，开展首届教育突出贡献奖评选表彰活动。公立医院改革进展顺利，分级诊疗稳步实施，医联体建设取得初步成效，“桃江模式”成为全省深化医疗体制改革名片。全面两孩政策有效落实。国家运动健康城市创建积极推进，奥林匹克公园获评全国体育旅游示范基地，承办湖南省第八届全民健身节等重大赛事。文物保护利用工作取得明显成效，益阳花鼓文化艺术创新工程荣获省首届文化创新奖。益阳烈士纪念广场顺利建成。社会大局和谐稳定。深入开展安全生产两大主题活动和系列专项整治行动，成功处置“5.11”蓝田隧道液化天然气大型半挂车侧翻事故，全市生产经营性安全事故、死亡人数、直接经济损失同比大幅下降，没有发生较大及以上生产安全责任事故，安全生产形势总体平稳向好。强化综治领导责任制，平安益阳建设持续深化，天网工程建设稳步推进，社会治安防控体系不断完善。全民普法深入开展，建成四级公共法律服务平台。推行公开接访、主动下访、依法治访，着力化解一批重点领域矛盾问题和信访积案。安全生产和综治工作获评全省先进单位。

六、着力推进自身建设，服务水平有效提升

政务服务持续优化。“放管服”改革取得实效，“四单一网”全面建立，便民服务体系改革被确定为全省十大改革创新经验之一。全力推进新型智慧城市建设，荣获“2017中国智慧城市创新奖”。“互联网＋政务服务”深入推进，服务效能不断提高。依法行政全面推进。认真落实领导干部学法制度和政府常务会议法治专题讲座制度。出台《益阳市行政执法监督暂行办法》，严格规范行政执法。规范性文件合法性审查全面落实。自觉接受市人大及其常委会法律监督、工作监督和市政协民主监督，认真办理人大代表建议和政协委员提案，办理质量和代表、委员满意率进一步提高。廉政建设不断加强。落实全面从严治党主体责任，切实履行“一岗双责”。着力解决发生在群众身边的不正之风和腐败问题，严肃查处了一批民生保障、脱贫攻坚、环境保护等领域的违纪违法案件。推行“互联网＋监督”，强化审计监督，开展“中梗阻”问题整治，政风行风进一步好转。

国防动员、武警、消防、人防、民兵预备役和军民融合深度发展等工作不断加强。工商行政管理、质量技术监督和食品药品监管不断强化。统计、档案、保密、对台、科协、烟草、地震、气象、水文、地方志、民族宗教、供销合作、住房公积金、公共资源管理等工作不断进步；工会、共青团、妇联等群团组织在经济社会发展中发挥了积极作用。

特别值得一提的是，2017年我们面对历史罕见的洪涝灾害，科学调度，万众一心，顽强拼搏，经受了12轮强降雨袭击，打赢了山洪防御、水库防守、城市防洪、堤垸保安四大保卫战，战胜了小河口溃垸性特大险情，创造了工程抢险“千分之一的奇迹”，最大限度保卫了人民群众生命财产安全，夺取了抗洪救灾全面胜利。得到了省委、省政府的充分肯定，赢得了人民群众的由衷赞扬！

各位代表，2017年是我们砥砺奋进、团结拼搏、成果丰硕的一年。盘点2017，成绩来之不易，这是以习近平同志为核心的党中央坚强领导的结果，是省委、省政府和市委坚强领导的结果，是市人大、市政协监督支持的结果，是全市上下共同努力的结果。在此，我谨代表市人民政府，向全市人民，向各民主党派、工商联、无党派人士、各人民团体，向驻益人民解放军和武警官兵、政法干警，向中央、省属驻益单位，向热心支持益阳经济社会发展的社会各界人士，表示衷心的感谢并致以崇高的敬意！

在总结成绩的同时，我们也清醒地看到，当前还存在许多问题和不足，与人民群众的期待还有不小差距。主要是：产业规模不大、结构不优，经济发展质量效益亟待提升；教育、卫生等重点民生领域短板仍然突出；重大风险防控、脱贫攻坚和污染治理的任务仍然十分繁重；创新氛围不浓，开放步伐不快，改革力度和深度仍需进一步加大；办事效率不高、投资环境不优的状况仍未根本改变，干部思想观念和能力素质与新常态、新理念、高质量发展要求不相适应，自身建设仍需进一步加强，等等。我们必须直面这些矛盾和问题，采取有力措施，切实加以改进。

2018年工作安排

2018年是贯彻党的十九大精神的开局之年，是改革开放40周年，是决胜全面建成小康社会、实施“十三五”规划承上启下的关键一年。2018年政府工作的指导思想是：全面贯彻党的十九大精神，以习近平新时代中国特色社会主义思想为指导，坚持党对经济工作的领导，坚持稳中求进工作总基调，坚持新发展理念，紧扣社会主要矛盾变化，按照高质量发展的要求，统筹推进“五位一体”总体布局和协调推进“四个全面”战略布局，以供给侧结构性改革为主线，突出新时代益阳新发展主题，深入实施产业兴市、创新引领、开放崛起、乡村振兴、可持续发展战略，聚焦聚力园区经济发展、产业项目建设、综合交通建设，统筹做好稳增长、促改革、调结构、惠民生、防风险各项工作，打好防范化解重大风险、精准脱贫、污染防治攻坚战，着力抓重点、补短板、强弱项、守底线，努力实现更高质量、更有效率、更加公平、更可持续的发展。

2018年经济社会发展的主要目标是：生产总值增长8.5%左右，固定资产投资增长13.5%，规模工业增加值增长8%，社会消费品零售总额增长11%，一般公共预算收入增长8.5%，城乡居民收入增长与经济增长基本同步，城镇登记失业率控制在省定目标以内，主要污染物排放总量明显减少。全社会研发投入占地区生产总值比重、制造业投资占固定资产投资比重、税收占一般公共预算收入比重明显提升。

完成上述目标任务，必须突出抓好以下九个方面工作：

一、强力推进新型工业化，发展壮大实体经济

扎实开展“产业项目建设年”活动，实施产业兴市战略，加快产业转型升级，提升新型工业化发展水平。一是突出抓好产业链建设。按照主攻优势产业、突出新兴产业、提升传统产业的思路，强化政策措施，重点发展先进轨道交通装备、工程机械、新型轻合金和碳基材料、生物医药、装配式建筑、农业机械、新能源装备、电子信息、食品、船舶制造等10大特色优势产业链，在强链、补链、延链、建链上取得实质性突破。实施“五个10”工程，抓好产业项目策划、论证、申报，争取更多产业项目进入全省“五个100”项目库。围绕产业链建设实施精准招商，着力引进一批发展前景好、带动能力强的重大产业项目，狠抓项目落地、开工、竣工、投产等关键环节，拉长产业链，提高集聚度，提升竞争力。二是持续推进园区建设大会战。理顺园区管理体制机制，进一步向园区放权，充分释放园区发展活力，支持引导园区差异化、特色化发展。推动益阳高新区加快发展。继续实施园区建设“四百工程”，完成基础设施投资100亿元、新建标准化厂房100万平方米、新开工项目100个、新投产项目100个以上，标准化厂房利用率达80%以上，园区单位面积投资强度、规模工业增加值、工业税收和工业集中度明显提升。推动产城融合发展，加强园区水、电、路、气、网等基础设施建设，增强住房、医疗、教育、文化、商贸、公交等综合服务供给能力，发展现代物流、仓储、中介、管理咨询等生产性服务业，着力提升园区承载能力。三是深入开展企业帮扶行动。重点帮扶100家以上成长性好、有市场竞争力的企业。持续开展金融服务创新示范园建设和政银企担对接活动，充分发挥创业投资基金的引导作用，推进过桥资金、风险保证金和担保体系建设。着力优化发展环境，积极构建“亲”“清”新型政商关系，及时帮助企业解决融资、用地、用工等难题。严格规范涉企检查，继续清理涉企收费，让企业在降成本上有切身感受。四是培育壮大现代企业。坚持把推进企业上市作为发展壮大实体经济、促进产业转型升级的战略性举措，加大企业上市支持奖励力度，鼓励企业进入资本市场做大做强。贯彻落实《中小企业促进法》，加强中小企业服务平台建设。引导企业建立和完善现代管理制度。大力弘扬劳模精神和工匠精神，着力建设知识型、技能型、创新型劳动者大军。大力弘扬企业家精神，尊重企业家和纳税人，不断激发企业发展活力和内生动力。

二、大力实施乡村振兴战略，推进农业农村现代化

按照“产业兴旺、生态宜居、乡风文明、治理有效、生活富裕”总要求，大力推进农业供给侧结构性改革，扎实抓好国家现代农业示范区建设和省级现代农业综合改革试验。一是推动农村一二三产业融合发展。全面落实粮食安全责任制，优化调整粮食生产结构，大力推广稻虾、稻鱼、稻蟹等生态种养模式，全面提升粮食生产质量效益。大力推进“三园”建设，深入实施“农业绿色发展五大行动”和现代农业“131千亿级产业”工程，深化农产品精深加工，发展休闲农业、智慧农业、农村电子商务等新型业态，带动农民增收致富。举办第二届生态农业智慧乡村互联网大会，推动“互联网＋现代农业”持续发展。二是加强农业品牌建设。全面推进“三品”工程，不断增强安化黑茶、桃江笋竹、赫山兰溪大米、资阳休闲食品、沅江芦笋、南县小龙虾、大通湖蟹等品牌影响力，推动“一县一品”特色发展，提升绿色生态农产品供给能力，构建农产品质量安全追溯体系，提高农产品质量安全水平，着力打造优质农副产品供应基地。三是培育发展新型农业经营主体。深化农村集体产权制度改革，落实承包地“三权”分置制度，推动农村土地规范有序流转，积极培育新型职业农民。实施新型农业经营主体培育工程，新增市级示范家庭农场和农民合作示范社各15家、省级龙头企业10家。四是推进美丽乡村建设。推进村庄规划编制全覆盖，加快农村村民住房建设管理立法。实施农村清洁工程，推动村庄美化、净化、绿化、亮化、序化、信息化，不断改善农村人居环境。推进乡村振兴示范点（片）和田园综合体建设，新创建5个以上省级美丽乡村示范村。进一步引导乡村移风易俗，破除陈规陋习，倡导文明新风，健全自治、法治、德治相结合的乡村治理体系，提高乡村治理能力和水平。

三、着眼扩量提质增效，大力发展现代服务业

推动服务业向高端化、智能化、特色化发展，提升现代服务业质量效益。一是加快发展全域旅游。强化规划引领，统筹推进休闲益阳、运动益阳、健康益阳建设，推动医养文体旅融合发展。加强旅游品牌推广和旅游产品包装策划，加快茶乡花海、桃花江旅游度假区等重点旅游项目和旅游基础设施建设。办好第十届竹文化节、第四届黑茶文化节和第二届涂鸦艺术节等活动，持续提升“益山益水、益美益阳”知名度和美誉度。二是加快发展文化产业。健全现代文化产业体系，培育新型文化业态。举办第二届中国（湖南）花鼓文化艺术节，进一步挖掘花鼓戏等特色文化资源，打造文化产业品牌。大力推进湖南工艺美术创意设计产业园、明清古巷文化旅游综合体等项目建设。三是加快发展现代金融。以金融服务实体经济为导向，优化信贷结构，推进普惠金融，加大中小微企业金融服务力度。进一步支持现有金融机构的发展，推进非银行类金融机构建设，组建市级国有融资性担保公司。发挥保险补偿稳定功能，扩大农业保险等政策性保险覆盖范围，提高保险密度和深度。四是加快发展电子商务。依托优势资源和产业，以中南电商园和中小企业电子商务孵化园为平台，加快本土企业及品牌培育。加强校企对接，支持市内高校开展电子商务学科建设，培养电子商务人才。进一步发挥“益村”平台作用，加快电子商务进农村。五是加快发展现代商贸物流。着力完善市县乡村四级物流体系，支持“快递下乡”。推进南洲物流园二期、桃江现代商贸物流园等项目建设。促进商贸业发展，扶持现有限额以上企业做大做强，着力培育新的限上企业。六是促进房地产健康发展。牢牢把握“房子是用来住的，不是用来炒的”这一定位，合理调控规范房地产市场，加快建立多主体供给、多渠道保障、租购并举的住房制度，努力实现房地产高端有供给、中端有市场、低端有保障。

四、实施创新引领开放崛起战略，持续激发发展活力

当今时代，惟创新者进，惟开放者强，惟改革者胜。一是全面深化改革。贯彻落实全国、全省整体部署，坚持以经济体制改革为重点，确保各项改革落到实处。深化“放管服”改革。加快推进政府融资平台转型发展。深化商事制度改革，全面推行“证照分离”。深化教育、文化、金融、国资监管、环境监管、供销合作等重点领域改革，不断释放改革红利，促进动力变革。二是持续扩大开放。强化招商引资“三个第一”意识，推进“五大开放行动”，确保引进市域外资金形成固定资产投资增长15%以上。举办第二届“汇智聚力·建设益阳”恳谈会，吸引益阳籍人士返乡投资兴业。大力扶持外向型企业发展，支持企业开展对外贸易与合作，积极参与“湘企出海”行动，继续实施“破零倍增”计划，外贸进出口额增长16%以上。加强与国外友好城市交流合作，推动中以合作示范城市建设，不断提高开放度。三是着力推进创新。加大科技投入，搭建创新平台。实施创新企业培育计划，新增高新技术企业10家以上，高新技术产业增加值占地区生产总值比重达到20%以上。继续举办企业创新创业大赛，鼓励引导企业开展产学研合作，推进院士产业园建设，努力提高科技成果转化率。加强知识产权保护和运用，确保有效发明专利增速高于全省平均水平。健全人才工作体制机制，创新人才引进方式，加大人才引进力度，提升服务人才水平，让各类人才的创造力竞相迸发、聪明才智充分涌流。

五、着力提升功能品质，加快推进新型城镇化

坚持走以人为核心的新型城镇化道路，提升城镇治理能力和发展水平。一是加快推进益沅桃城镇群建设。扎实开展益沅桃城镇群产城融合试点，突出基础设施互联互通、区域产业互补发展、生态环境协同治理、公共服务融合提升，增强益沅桃城镇群综合实力和竞争力。坚持不懈以全国文明城市创建为总揽，统筹各项创建工作，着力提升城市品位和市民素质。启动新一轮城市总规修编和相关专项规划编制，提升规划工作系统性和信息化水平。加快推进资江风貌带、一园两中心、城市西环线等重大项目建设。突出城市“双修”，实施“三微”项目建设二期工程。完善城区路网结构，完成海棠中学、市一中等重要节点过街通道建设，打通团圆南路、梅林路等城市道路。推进海绵城市建设，实施中心城区“排水防涝三年行动”计划，完成城市黑臭水体整治年度任务。深化城市管理执法体制改革，强力开展控违拆违专项行动，强化规划执行刚性约束。进一步规范中心城区交通秩序。二是强力推进东接东进。进一步加强与长沙在交通、产业等方面的对接，推进G319复线、港煤大道等重点项目建设，加强工业园区交流与合作，积极承接产业转移，发展配套产业，实现互补发展。统筹东部片区基础设施、公共服务和商务配套项目规划建设。三是着力打造新型智慧城市。紧扣“善政、惠民、兴业”总目标，按照顶层设计要求，创新投融资方式，积极推进相关项目建设。以平台为基础，以数据为纽带，深化与华为等龙头企业合作，引进华强聚丰等产业互联网企业，着力发展大数据、云计算、物联网等新兴产业，加快发展数字经济。四是着力推进县城和重点镇建设。实施县城扩容提质工程，加快城乡供水、供气、污水处理和公交一体化步伐，重点推进天然气加气站、城镇雨污分流管网、污水处理厂建设，提升县城综合承载能力和公共服务水平。推进西部和北部两个垃圾焚烧发电项目建设，加快城乡垃圾一体化处理全覆盖进程。加强重点镇、特色镇建设，着力培育一批工业强镇、商贸重镇、旅游名镇。

六、坚持人与自然和谐共生，奋力推进生态文明建设

牢固树立和大力践行绿水青山就是金山银山的理念，像保护眼睛一样保护生态环境，像对待生命一样对待生态环境，坚定不移走生产发展、生活富裕、生态良好的发展之路。一是全面推进绿色发展。发展绿色低碳循环经济，壮大节能环保、清洁生产、清洁能源产业。加快生产方式绿色化进程，推进传统产业绿色化升级改造，提高能源资源利用效率，减少污染物排放。践行低碳生活理念，倡导绿色健康生活方式。抓好两型标志性工程和县级两型综合示范区建设。二是坚决打好污染防治攻坚战。坚持以河长制为总抓手，全面推行“三长一站”模式，加大“一河一策”“一湖一策”工作落实力度，统筹推进水环境综合治理。启动洞庭湖生态环境专项整治和资江锑污染整治三年行动计划，抓好河道采砂、水上运输和工业污染整治及生态修复工作。新建48个乡镇污水处理厂。建立大气污染防治长效机制，强化燃煤锅炉淘汰改造、餐饮油烟和建筑工地扬尘治理、农村秸秆禁烧等工作，确保城区空气质量持续改善。加强土壤污染防治，抓好土壤环境监测预警、分类管控、治理修复，强化重金属污染防治和危险废物监管。做好全国第二次污染源普查工作。狠抓中央和省环保督察反馈问题整改，确保生态环境质量不断优化。三是加大生态系统保护力度。落实国省主体功能区规划，科学划定生态保护、永久基本农田、城镇开发边界三条控制线。强化土地集约节约利用。落实城市规划区山体水体保护“一条例一规划一办法”，保持山水元素完整性，形成山水交融生态空间。加强南洞庭湖湿地保护，建立健全保护区管理体制机制，继续推进“百湖湿地”修复，完成缓冲区、实验区黑杨退出年度任务。深入实施退耕还林、长防林、天然林保护、石漠化治理和森林抚育工程，加强森林资源保护管理，确保森林覆盖率稳定在54.46%，确保国家森林城市复查顺利通过。

七、加快推进基础设施建设，着力夯实发展基础

加速补齐基础设施短板，不断改善发展条件。一是强力推进交通建设大会战。完成交通建设投资158亿元。加快长益复线、官新、伍益、宁韶高速公路建设，确保南益、马安高速公路建成通车，确保实现“县县通高速”目标。创新干线公路PPP投融资模式，加快实施“519”行动，完成西流湾大桥等10个重点项目建设，推进南益高速大通湖连接线等31个项目建设，实现境内高速公路落地互通与干线公路、农村公路联网对接。完成农村公路加宽610公里、危桥改造117座，建设通自然村公路1431公里。全力支持常益长高铁建设，扎实做好轨道交通和通用机场建设前期工作。二是加强农田水利建设。加大农村土地综合整治力度，抓好高标准农田建设。完成资阳、南县、沅江、桃江等4个中央财政农田水利项目县建设工作。完成水利建设投资34亿元，重点实施水毁恢复、堤防加固、水库水闸除险加固等水利工程，统筹抓好洞庭湖北部地区分片补水工程建设，大幅提升防洪抗旱能力，实现“五个确保”目标。三是不断夯实信息发展基础。推进电信普遍服务，加强通信基础设施共建共享。加快信息化应用进村入户，力争全市行政村光纤通达、4G网络基本实现全覆盖。启动5G网络规划建设。四是加强能源基础设施建设。抓好农网升级改造和毛家塘变电站改造等城网建设，推进光伏发电、生物质能发电等新型能源建设。做好金塘冲水库建设相关工作。完成“气化湖南”工程益阳境内管线建设目标。支持电动汽车充电基础设施等项目建设。侯卫东官场笔记

八、坚持以人民为中心，着力保障和改善民生

人民对美好生活的向往，就是我们的奋斗目标。始终坚持民生至上，多谋民生之利，多解民生之忧。一是坚决打好脱贫攻坚战。扭住产业扶贫这个关键，扎实推进“六个一批”“六大建设”，深入开展“万企帮万村”“互联网＋社会扶贫”工作，坚持扶贫同扶志、扶智相结合，激发贫困人口内生动力。强化扶贫资金监管，确保每一分钱都用在刀刃上、用到人民群众心坎上。完成易地扶贫搬迁、扶贫小额贷款发放年度目标任务，确保9.5万贫困人口脱贫、119个贫困村退出，确保国家扶贫开发工作重点县安化县摘帽。二是加快建设教育强市。把教育事业放在优先位置，努力办好人民满意的教育。促进学前教育公益普惠，实现乡镇公办幼儿园全覆盖。启动县乡教育布局规划，全面推进县域内城乡义务教育一体化改革发展。改造薄弱学校39所，建设合格学校54所。推进化解大班额三年行动计划，2018年基本消除66人以上超大班额。推动普高优质多样发展，教育质量在全省争先进位。完善职业教育和培训体系，深化产教融合、校企合作。促进民办教育健康发展。支持湖南城市学院加快“双一流”建设，推动益阳师范申专工作。突出抓好学校安全工作，强化校车、消防、校园及周边环境、食品、饮用水、公共卫生等安全监管，努力让学生安心、让家长放心。三是着力提升就业质量。落实积极就业政策，确保新增城镇就业3.4万人。突出抓好高校毕业生、城镇就业困难对象等重点群体就业，继续保持就业帮扶动态“双清零”。加强农民工和贫困家庭“两后生”就业技能培训。大力推进大众创业万众创新，落实创业优惠政策，鼓励创业带动就业。四是加强社会保障体系建设。不断扩大社会保险覆盖面。完善基本医疗保险异地直接结算，实现城乡居民大病保险市级统筹。加强困难群体救助，推进城乡低保统筹发展和特困人员救助供养。完成10所敬老院改扩建，积极推进移民避险解困。推进保障性住房建设，加快城中村改造，完成城镇棚户区和农村危房改造年度任务；强化公租房分配管理，着力提升入住率，确保公租房建得好、分得公、住得进。五是推进“健康益阳”建设。深化医药卫生体制改革，推进医联体建设和县级公立医院综合改革及示范创建工作，抓好分级诊疗和家庭医生签约服务。加大乡村医生本土化培养力度，加强村卫生室和社区卫生服务体系建设。推动中医药事业发展。深入开展爱国卫生运动，提高群众公共卫生意识。坚持卫生实用标准，扎实推进“厕所革命”。完善传染病监测预警和联防联控，严密防控公共卫生事件发生。抓好农村适龄妇女“两癌”免费检查工作。推进医养结合，支持康雅医院创建全国医养结合示范项目，探索医养联合体新模式；加快居家养老服务平台建设。落实两孩配套政策，促进人口科学发展。加强残疾康复服务，建好市残疾人康复中心。落实“四个最严”要求，强化食品药品监管和专项整治，确保人民群众“舌尖上的安全”。六是大力发展文化体育事业。加快“运动益阳”建设，推进国家运动健康城市、体育强县、运动休闲特色小镇创建工作，完善基本公共体育设施。加强公共文化服务体系建设，推进七大文化惠民工程。繁荣文艺创作，提供更多群众喜闻乐见的文化产品。加快万里茶道联合申遗工作，争取兔子山遗址、梅城文武庙获批国家级文物保护单位。七是维护社会安定和谐。打好防范化解重大风险攻坚战，全面防范各类风险发生。继续开展安全生产两大主题活动，不断完善安全隐患治理“一单四制”，稳步提高安全生产监管能力和企业安全生产水平，坚决守住安全生产底线。积极化解政府性债务，加强风险预警处置，严守政策底线，逐步消化存量，严格控制增量。优化金融生态环境，严厉打击非法集资、高息揽储等违法违规金融活动，坚决守住不发生区域性、系统性金融风险底线。健全“信访工作村（社区）为主”机制，深入开展“信访法治建设年”和“民情大走访”活动，依法及时就地解决信访问题。健全公共法律服务体系，加大法律援助力度，维护群众合法权益。实施守信联合激励和失信联合惩戒，推进诚信益阳建设。依法管理民族宗教事务，助推民族乡村经济社会发展。扎实推进雪亮工程、天网工程、消防基础设施和综治中心建设，切实加强农村交通管理，深化网格化服务管理，推动平安创建工作向纵深发展。严厉打击黄赌毒、黑拐骗等违法犯罪活动，保障群众生命财产安全，增强群众安全感。

抓好国防动员、兵役征集、“双带双促”和军民融合深度发展工作。支持主流媒体做优做强。支持工会、共青团、妇联等群团组织开展工作。加强留守儿童保护，关爱青少年健康成长。扎实做好全国第四次经济普查准备工作。统筹推进国家安全、对台、人防、统计、地方志、档案、保密、气象、水文、烟草、住房公积金等各项工作。

九、牢记为民服务宗旨，建设人民满意政府

完成奋斗目标，实现美好蓝图，必须全面加强政府自身建设，不断提高为人民服务的能力和水平。一是推进高效政府建设。大兴调查研究之风，大兴学习之风，深入推进“两学一做”学习教育常态化制度化，扎实开展“不忘初心，牢记使命”主题教育，强化“四个意识”，增强“四个自信”，提升“八种执政本领”。加快“两集中两到位”，大力推进“互联网＋政务服务”，不断创新和优化网上服务事项和流程，让数据多跑路、群众少跑腿。全面落实省委省政府《对接“北上广”优化大环境行动导则》，不断优化经济发展环境，进一步缩短涉企行政审批及服务事项办理时限。完善常态化督查机制，严格绩效管理考核，开展重大政策执行绩效评估，确保各项决策部署落实到位。坚持激励约束双向发力，大力整治不作为、乱作为，深化“中梗阻”专项治理，严厉查处工作落实不力、效能低下、敷衍塞责、弄虚作假等行为。旗帜鲜明地为那些敢于担当、踏实做事、不谋私利的干部撑腰鼓劲，激励干部雷厉风行、干事创业。二是推进法治政府建设。全面推进法治益阳建设，坚持依法行政，严格按程序决策、按规矩办事，确保政府工作经得起法律的检验。依法接受市人大及其常委会的法律监督和工作监督，执行各项决议、决定，落实规范性文件备案审查。自觉接受市政协民主监督，认真听取各民主党派、工商联、无党派人士和人民团体意见。认真办理人大代表建议和政协委员提案，创新办理方式，增强办理实效。全面推进政务公开，完善重大行政决策机制。进一步规范行政执法行为，落实法律顾问制度，不断提高运用法治思维和法治方式深化改革、推动发展、化解矛盾、维护稳定的能力和水平。三是推进廉洁政府建设。保持政治定力，以反腐败永远在路上的坚韧和执着，坚持把纪律和规矩挺在前面，认真履行“一岗双责”，严格落实党风廉政建设和作风建设各项规定，加强廉政风险防控，驰而不息纠正“四风”。推进审计监督全覆盖，规范公共资源交易，让权力在阳光下运行。坚持有腐必反、有贪必肃，坚决铲除滋生腐败的土壤，坚决消除权力寻租的空间，坚决斩断伸向群众利益的“黑手”，努力营造风清气正的政治生态，确保干部清正、政府清廉、政治清明。

各位代表，潮起海天阔，扬帆正当时。开启新征程，推动新发展，建设新益阳，是新时代赋予我们的神圣职责和光荣使命。让我们高举习近平新时代中国特色社会主义思想伟大旗帜，深入贯彻落实党的十九大精神，在省委、省政府和市委的坚强领导下，团结依靠全市人民，以滚石上山的气魄和踏石留印的作风，为实现人民对美好生活的向往，为建设富饶创新开放绿色幸福新益阳而努力奋斗！
